# An ontology of mechanisms of action in behaviour change interventions

**DOI:** 10.12688/wellcomeopenres.19489.1

**Published:** 2023-08-11

**Authors:** Paulina M. Schenk, Alison J. Wright, Robert West, Janna Hastings, Fabiana Lorencatto, Candice Moore, Emily Hayes, Verena Schneider, Susan Michie

**Affiliations:** 1 Centre for Behaviour Change, University College London, London, England, UK; 2 Institute of Pharmaceutical Science, King's College London, London, England, UK; 3 Department of Behavioural Science and Health, University College London, London, England, UK; 4 Institute for Implementation Science in Health Care, Universitat Zurich, Zürich, Zurich, Switzerland; 5 School of Medicine, University of St Gallen, St. Gallen, St. Gallen, Switzerland; 6 Research Department of Epidemiology and Public Health, University College London, London, England, UK

**Keywords:** intervention, ontology, behaviour, reporting, evidence synthesis, mechanism of action, mechanism of change, process of change, determinant

## Abstract

**Background:** Behaviour change interventions influence behaviour through causal processes called “mechanisms of action” (MoAs). Reports of such interventions and their evaluations often use inconsistent or ambiguous terminology, creating problems for searching, evidence synthesis and theory development. This inconsistency includes the reporting of MoAs. An ontology can help address these challenges by serving as a classification system that labels and defines MoAs and their relationships. The aim of this study was to develop an ontology of MoAs of behaviour change interventions.

**Methods:** To develop the MoA Ontology, we (1) defined the ontology’s scope; (2) identified, labelled and defined the ontology’s entities; (3) refined the ontology by annotating (i.e., coding) MoAs in intervention reports; (4) refined the ontology via stakeholder review of the ontology’s comprehensiveness and clarity; (5) tested whether researchers could reliably apply the ontology to annotate MoAs in intervention evaluation reports; (6) refined the relationships between entities; (7) reviewed the alignment of the MoA Ontology with other relevant ontologies, (8) reviewed the ontology’s alignment with the Theories and Techniques Tool; and (9) published a machine-readable version of the ontology.

**Results:** An MoA was defined as “a process that is causally active in the relationship between a behaviour change intervention scenario and its outcome behaviour”. We created an initial MoA Ontology with 261 entities through Steps 2-5. Inter-rater reliability for annotating study reports using these entities was α=0.68 (“acceptable”) for researchers familiar with the ontology and α=0.47 for researchers unfamiliar with it. As a result of additional revisions (Steps 6-8), 21 further entities were added to the ontology resulting in 282 entities organised in seven hierarchical levels.

**Conclusions:** The MoA Ontology extensively captures MoAs of behaviour change interventions. The ontology can serve as a controlled vocabulary for MoAs to consistently describe and synthesise evidence about MoAs across diverse sources.

## Introduction

Behaviour change interventions can operate at an individual- and population-level to improve health, wellbeing and environmental sustainability ( Ayouni *et al.*, 2021; Funk *et al.*, 2010; National Institute for Clinical Excellence, 2007; Swim *et al.*, 2011; van Valkengoed & Steg, 2019). There are nine general types of interventions aimed at changing behaviour, summarised in the Behaviour Change Wheel framework ( Michie *et al.*, 2011): education, persuasion, incentivisation, coercion, training, enablement, modelling, environmental restructuring and restriction. However, intervention effectiveness varies widely ( Nielsen *et al.*, 2018). One way of improving the effectiveness of interventions is to understand “why” interventions change behaviours, that is their processes of change or “mechanisms of action” (MoAs) ( Hardeman *et al.*, 2005; Michie & Abraham, 2004; Nielsen *et al.*, 2018).

**Figure 1.**
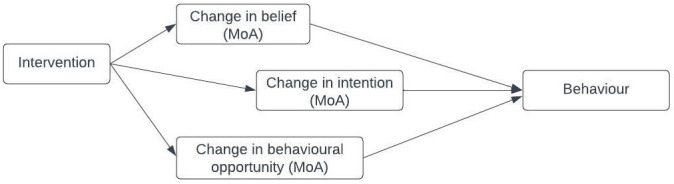
Schematic representation of the links between an intervention, its MoAs and target behaviour.

MoAs have been defined as “the type(s) of process by which interventions influence the target behaviour” ( Michie *et al.*, 2017). An example of the relationship between an intervention, its MoAs and a target behaviour is shown in Figure 1: an intervention influences behaviour through beliefs, intentions, and behavioural opportunities. Investigating and synthesising evidence about whether an intervention has influenced an MoA, and thereby the target behaviour, helps to explain why an intervention was, or was not, effective ( Nielsen *et al.*, 2018). For instance, systematic reviews of physical activity interventions showed that changing beliefs about capabilities (an MoA) was frequently associated with increases in exercising ( Bauman *et al.*, 2012). Intervention developers can use this evidence to design interventions to increase physical activity in similar contexts by targeting beliefs about capability; such interventions are more likely to change the target behaviour than interventions designed without this knowledge.

Theories of behaviour specify modifiable constructs (e.g., beliefs) and other less modifiable/unmodifiable constructs (e.g., age, past experience) that influence behaviour ( Davis *et al.*, 2015; Eccles *et al.*, 2005). Modifiable theoretical constructs can guide which MoAs should be targeted to change specific behaviours ( Collins *et al.*, 2011; Glanz & Bishop, 2010; Sheeran *et al.*, 2017). However, there are well over 80 theories of behaviour and behaviour change ( Davis *et al.*, 2015; Michie & Johnston, 2012). Many of these propose potential MoAs that overlap, or share a label but have different definitions or have the same definition but not the same labels ( Sheeran *et al.*, 2017). For example, the Integrative Model of Health Attitude and Behaviour Change ( Flay, 1981) and the Theory of Interpersonal Behaviour ( Triandis, 1977) include two potential MoAs with different labels: “expectancy” and “perceived consequence”. However, both MoAs are defined as a belief about the likely outcomes of behaviour. Reports of intervention evaluations therefore often use labels, definitions and measurements for MoAs inconsistently ( Abraham *et al.*, 2014; Carey *et al.*, 2019; Nielsen *et al.*, 2018; Prestwich *et al.*, 2014; Prestwich *et al.*, 2015). Without a common shared vocabulary, there are challenges for understanding, comparing and synthesising evidence about MoAs across intervention reports, limiting our ability to accumulate knowledge about how behaviour change interventions have their effects ( National Academies of Sciences, 2022; Noar & Zimmerman, 2005).

In other scientific fields (e.g., biomedicine), ***ontologies*** have helped create a shared language and thereby organised complex knowledge ( Gene Ontology Consortium, 2015; Larsen *et al.*, 2017; Norris *et al.*, 2019) (see glossary of bold, italicised terms in Table 1). An ontology is a classification system that includes representations of entities (anything that exists, such as objects, processes or roles) with clearly expressed labels for each entity, unambiguous definitions, and the relationships between entities ( Arp *et al.*, 2015; Michie *et al.*, 2017). Note that entities in ontologies can also be referred to as classes, but for simplicity we will use the term “entity” throughout. Entity definitions and ***relationships*** are specified using a logic-based language and unique identifiers, making ontologies accessible to computers ( Hastings, 2017; Seppälä *et al.*, 2014).

**Table 1.** Glossary of terms.

Term	Definition	Source
Annotation	Process of coding selected parts of documents or other resources to identify the presence of ontological entities.	Michie *et al.* (2017)
Annotation guidance manual	Written guidance on how to identify and tag pieces of text from intervention evaluation reports with specific codes relating to entities in the ontology, using for example EPPI- Reviewer software.	Michie *et al.* (2017)
Behaviour change technique	A planned process that is the smallest part of BCI content that is observable, replicable and on its own has the potential to bring about behaviour change.	Marques *et al.* (2023)
Basic Formal Ontology (BFO)	An upper-level ontology specifying foundational distinctions between different types of entity, such as between continuants and occurrents, developed to support integration, especially of data obtained through scientific research.	Arp *et al.* (2015)
Class	Classes in ontologies represent types of entities in the world. The terms “entity” and “class” can be used interchangeably to refer to the entities represented in an ontology. Classes can be arranged hierarchically by the specification of parent and child classes; see definition of parent class in the glossary	Arp *et al.* (2015)
Continuant	An entity that continues to exist as the same individual over time, for example, objects and spatial regions.	Arp *et al.* (2015)
Domain-neutral entity	A very broad entity that is relevant to a broad range of scientific domains, rather than any particular domain, e.g., continuant and process. The entities in Basic Formal Ontology are domain neutral.	ISO/IEC 21838 (2021)
Entity	Anything that exists, including objects, processes, and their attributes. According to Basic Formal Ontology, entities can be broadly divided into continuants and occurrents.	Arp *et al.* (2015)
EPPI-Reviewer	A web-based software program for managing and analysing data in all types of systematic review (meta-analysis, framework synthesis, thematic synthesis etc.) It manages references, stores PDF files and facilitates qualitative and quantitative analyses. It also has a facility to annotate published papers.	Thomas *et al.* (2010) EPPI-Reviewer 4: http://eppi.ioe.ac.uk/eppireviewer4/ EPPI-Reviewer Web Version: https://eppi.ioe.ac.uk/eppireviewer-web/
GitHub	A web-based platform used as a repository for sharing code, allowing version control.	https://github.com/
Inter-rater reliability	Statistical assessment of similarity and dissimilarity of coding between two or more coders. If inter-rater reliability is high this suggests that entity definitions and labels are being interpreted similarly by the coders.	Gwet (2014)
Interoperability	Two systems are interoperable if data coming from each system can be used by the other system. Note: An ontology is interoperable with another ontology if it can be used together with or re-uses parts from the other ontology.	http://www.obofoundry.org/principles/fp-010-collaboration.html
OBO Foundry	The Open Biological and Biomedical Ontology (OBO) Foundry is a collective of ontology developers that are committed to collaboration and adherence to shared principles. The mission of the OBO Foundry is to develop a family of interoperable ontologies that are both logically well-formed and scientifically accurate.	Smith *et al.* (2007); www.obofoundry.org/
OBO Foundry principles	Good practice principles of ontology development and maintenance intended as normative for OBO Foundry ontologies. Ontologies submitted to OBO Foundry are evaluated against them.	http://www.obofoundry.org/principles/fp-000-summary.html
Ontology	A standardised representational framework providing a set of entities for the consistent description (or “annotation” or “tagging”) of data and information across disciplinary and research community boundaries.	Arp *et al.* (2015)
Parent class	An entity within an ontology that is hierarchically related to one or more child classes (subclasses) such that all members of the child class are also members of the parent class, and all properties of the parent class are also properties of the child class.	Arp *et al.* (2015)
Process	Something that takes place over time.	Arp *et al.* (2015)
Relationship	The manner in which two entities are connected or linked.	Arp *et al.* (2015)
ROBOT	An automated command line tool for ontology workflows.	Jackson *et al.* (2019); http://robot.obolibrary.org
Uniform Resource Identifiers (URI)	A string of characters that unambiguously identifies an ontology or an individual entity within an ontology. Having URI identifiers is one of the OBO Foundry principles.	http://www.obofoundry.org/principles/fp-003-uris.html
Versioning	Ontologies that have been released are expected to change over time as they are developed and refined, leading to a series of different files. Consumers of ontologies must be able to specify exactly which ontology files they used to encode their data or build their applications and be able to retrieve unaltered copies of those files in perpetuity. Versioning is one of the OBO Foundry principles.	http://www.obofoundry.org/principles/fp-004-versioning.html
Web Ontology Language (OWL)	A formal language for describing ontologies. It provides methods to model classes of “things”, how they relate to each other and the properties they have. OWL is designed to be interpreted by computer programs and is extensively used in the Semantic Web where rich knowledge about web documents and the relationships between them are represented using OWL syntax.	https://www.w3.org/TR/owl2-quick-reference/

Ontologies can be applied to writing study protocols and reports by using entity labels and definitions to unambiguously refer to constructs. Ontologies can also be employed in evidence synthesis, by ***annotating*** (coding) study reports for the presence of ontology entities to be included in the synthesis ( Gene Ontology Consortium, 2015). Since ontologies are computer readable, ontology-based algorithms can be developed to automatically extract information from study reports, organise that information according to the ontology and use this to predict outcomes ( Hastings, 2017; Hastings & Schulz, 2012; Matentzoglu *et al.*, 2018; Norris *et al.*, 2019; Seppälä *et al.*, 2017). Ontologies are designed and expected to be updated over time, in line with user feedback or scientific developments in relevant fields ( He *et al.*, 2018). 

Ontologies such as the Gene Ontology have successfully developed a shared language and thereby organised complex knowledge in biomedicine ( Gene Ontology Consortium, 2015). The Human Behaviour-Change Project (HBCP; Michie *et al.*, 2017) has applied a similar approach to representing behaviour change interventions. One application was to represent theories of behaviour change in ontological terms ( Hale *et al.*, 2020a; West *et al.*, 2020; for more detail, see Discussion). This work aimed to reduce the ambiguity about theories that arises from underspecified, sometimes vague, definitions of constructs and relationships ( Davis *et al.*, 2015). Using a theory-neutral ontological approach enabled the comparison between and integration of theories ( Hale *et al.*, 2020a; West *et al.*, 2020); the final study to achieve the latter is currently in progress.

**Figure 2.**
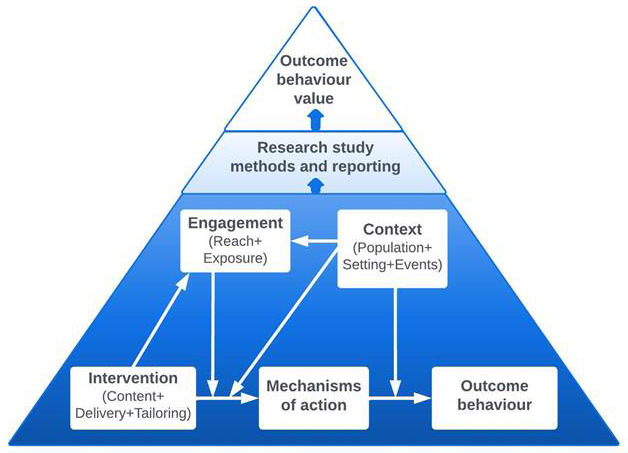
Schematic representation of the BCIO: key entities and causal connections.

The second application was to develop a formal, theory-neutral ontology to specify all the key aspects of a behaviour change interventions, the Behaviour Change Intervention Ontology (BCIO; Michie *et al.*, 2021). The BCIO includes key entities about behaviour change interventions and their evaluations. The top-level entities of the ontology are “behaviour change intervention (BCI) content”, “BCI engagement”, “BCI context” “BCI mechanism of action” and “outcome behaviour”, as shown in Figure 2. The MoA Ontology is the part of the BCIO which labels and defines key entities for MoAs in behaviour change interventions ( Michie *et al.*, 2017; Michie *et al.*, 2021; Wright *et al.*, 2020).

### Aim

To develop the MoA Ontology to serve as a clear, extensive and usable classification system to describe MoAs of behaviour change interventions.

## Methods

**Figure 3.**
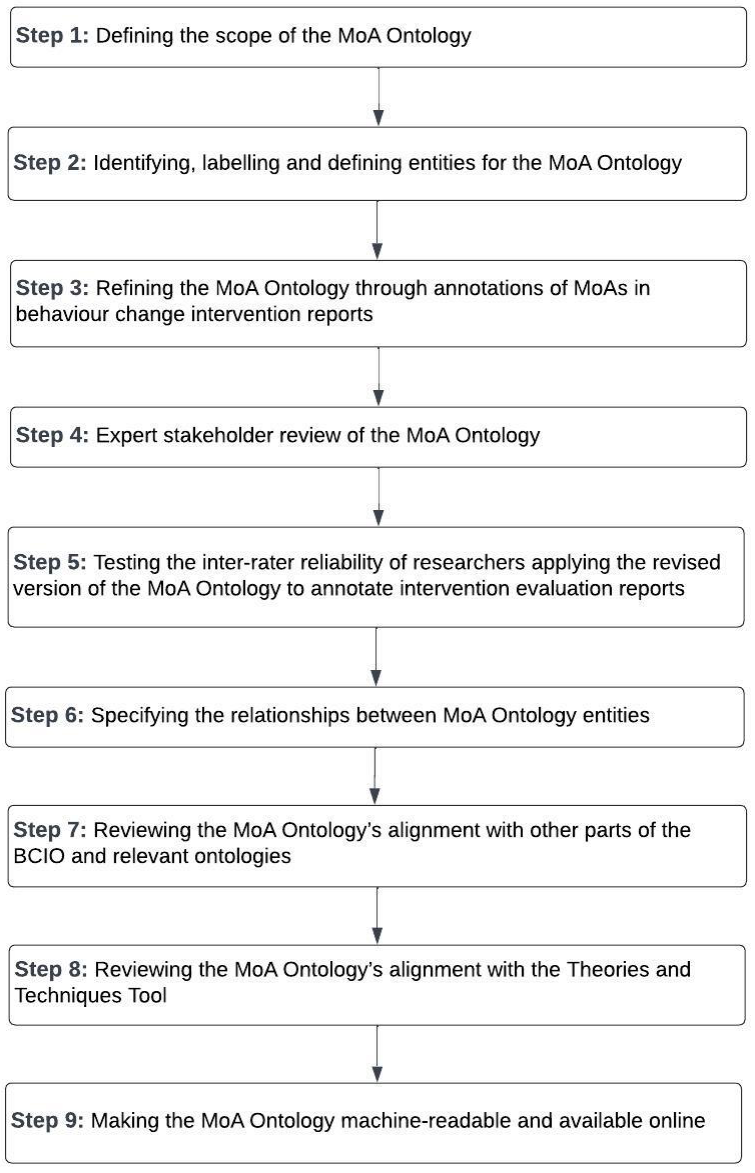
Overview of steps to develop the MoA Ontology.

The MoA Ontology was developed in nine broad steps using methods applied for other parts of the BCIO ( Wright *et al.*, 2020). Figure 3 presents an overview of these steps.

### Step 1 – Defining the scope of the MoA Ontology

To specify a scope for the MoA Ontology, the preliminary definition for an MoA was: “A process by which interventions influence the target behaviour” ( Michie *et al.*, 2017). This definition was refined during the later steps.

### Step 2 – Identifying, labelling and defining entities for the MoA Ontology

***Step 2.a - Identifying potential MoAs from behavioural theories***

To identify entities for the MoA Ontology, the starting point was 1733 constructs extracted from 83 theories identified in a scoping review of theories of behaviour and behaviour change ( Davis *et al.*, 2015). These constructs were labelled and defined based on their descriptions in the relevant theories or, where necessary, dictionaries. From these 1733 constructs, those that were considered changeable by an intervention and therefore could qualify as MoAs were identified by two researchers (CM & PS). To make these judgements, the two researchers independently applied criteria that had been iteratively developed (see Table 2 and additional guidelines in the link: https://osf.io/9j2be). For instance, from the Health Belief Model ( Rosenstock, 1974; Rosenstock *et al.*, 1988), the construct “perceived benefit” (definition: “Belief about the relative effectiveness of known options for reducing a health threat, distinct from objective facts.”) was judged changeable by an intervention and thus qualified as an MoA. The researchers compared their judgements and discussed disagreements and uncertainties, and where necessary, consulted three behavioural science experts (AW, SM & RW).

**Table 2.** Criteria for including or excluding a construct as a potential MoA.

Type of criteria	Criteria
Inclusion	Changes in momentary psychological and physiological states (e.g., fear, hunger or mood/arousal)
	Changes in manifestations of enduring psychological and physiological states or dispositions (e.g., cognitive ability, identity, or preparedness to change)
	Sequences of events transforming, or preventing transformation of states, stages or traits (e.g., habituation or associative learning)
	Changes in the physical and/or social environment where the theory specifies the influence on behaviour (e.g., physical/social opportunity, norm or interaction)
	Behaviours (e.g., avoidance behaviour)
Exclusion	A non-modifiable historical factor (e.g., prior experience or age)
	Only being changeable in a specific maturation period (e.g., tendency to respond to conflict physically which develops during maturation)
	Part of an intervention itself (e.g., a behaviour change technique)
	A target behaviour (e.g., physical activity with no influence specified on another behaviour)
	Including multiple processes and one or more of these are not mechanisms of action (e.g., process of teaching)

Drawing on the identified MoAs, the researchers independently coded MoAs that would qualify as compound MoAs, i.e., composites of distinct MoAs that would each work differently in interventions. Compound MoAs are not classifiable as a single entity and therefore were excluded from the ontology. For example, the MoA labelled “inner containment” was judged to qualify as a compound MoA based on its definition: “Factors involved in the regulation of the self, such as self-control, self-concept, the ability to tolerate frustration and resist diversions, etc” ( Reckless, 1961). Any constructs where the two researchers disagreed or were uncertain about whether the construct qualified as a compound MoA were discussed with the wider research team.

***Step 2.b - Grouping potential MoAs to identify candidate entities for the ontology***

From the 1733 theoretical constructs, some MoAs had been identified and the same or strongly overlapping MoAs were grouped in a study examining expert consensus about which behaviour change techniques might change which frequently occurring MoAs ( Connell *et al.*, 2019; Johnston *et al.*, 2018; Michie *et al.*, 2018). These preliminary MoA groups were updated according to the results from Step 2.a: constructs that were judged to no longer qualify as MoAs were removed from their previous groups. These updated groups served as candidate entities for the MoA Ontology.

Each MoA group was reviewed by at least two researchers (CM, EH & PS), as follows:

1. Read the labels and definitions of each MoA in a group and judged which attributes were shared by most MoAs in a group: examples of attributes are “negative affect” and “future-oriented”
2. Judged whether any MoAs did not share their groups’ attributes and so should be removed from the group and added to the pool of ungrouped MoAs

The researchers reviewed all ungrouped MoAs to judge whether they:

1. Shared any reviewed group’s attributes and so could be assigned to that group, or
2. Shared attributes with one or more other ungrouped MoA and so could form a new group

The labels and definitions of MoA groups were refined or created by reviewing the MoAs organised in each group. Any uncertainties or disagreements were discussed between the researchers and, where necessary, with the wider research team.

***Step 2.c - Identifying ontological entities based on MoA groups and reusing other ontologies where possible***

To identify unique entities from the MoA groups, two researchers checked each MoA group to see if it had at least two constituent MoAs with definitions that overlapped with the definitions of an entity from another relevant ontology. They searched existing ontologies for these entities using key terms (e.g., the MoA groups’ labels or synonyms) via specialist ontology databases, such as the Ontology Lookup Service ( European Bioinformatics Institute, 2019). Where there was a suitable entity in an existing ontology, it was used in the MoA Ontology. This practice reduces redundancy (i.e., unduplicated entities) and ensures ***interoperability*** between ontologies, in line with ***Open Biological and Biomedical Ontology (OBO) Foundry principles*** for developing “gold” standard ontologies ( OBO Foundry, 2019; Wright *et al.*, 2020). We reused entities from ontologies that: (1) conformed to the OBO Foundry’s technical principles, such as using ***Uniform Resource Identifiers*** (URIs) for entities ( Smith *et al.*, 2007) and (2) were structured using ***Basic Formal Ontology*** ( Arp *et al.*, 2015; Wright *et al.*, 2020), which contains broad ***domain-neutral entities***, such as ***continuants*** (e.g., objects) and ***occurrents*** (e.g., processes). By drawing on the same upper-level ontology and following shared technical principles, the structures of different ontologies become better aligned and thereby more interoperable, and common technical tools for ontologies (e.g., Jackson *et al.*, 2019) can be used in workflows, enabling re-use ( Matentzoglu *et al.*, 2022; Wright *et al.*, 2020).

For MoA groups that did not overlap with entities in other ontologies, the researchers judged whether these groups qualified as unique entities in the MoA Ontology. To qualify as a unique entity, an MoA group needed to have attributes that differentiated it from other entities in the ontology. For relevant groups, new entities were created by revising their preliminary labels and definitions, based on recognised principles for writing “good” ontological labels and definitions ( Michie *et al.*, 2019; Seppälä *et al.*, 2017). The groups that did not qualify as unique entities were removed and their MoAs were ungrouped.

Next, the researchers judged whether any constituent MoAs had attributes that distinguished them from their grouping entity and so qualified as separate entities in the ontology. For entities that were reused from other ontologies, the researchers investigated whether these ontologies had subclasses that overlapped with any constituent MoAs. If so, these subclasses were reused. Otherwise, the researchers developed new entities for constituent MoAs that were judged to have unique attributes. Similarly, the researchers judged whether any ungrouped MoA had unique attributes that differentiated them from other entities in the MoA Ontology. For relevant ungrouped MoAs, the researchers searched for appropriate entities in other ontologies to reuse or developed new entities for the MoA Ontology.

All entities were reviewed by two researchers to ensure that the entities were sufficiently distinguished from one another and did not add excessive detail to the ontology. Entities that were not sufficiently distinguished from their parent class were removed from the ontology. Finally, the research team grouped and structured the MoA Ontology’s entities, specifying hierarchical relationships between entities where appropriate (e.g., “self-efficacy belief for a behaviour” is_a “belief”), and linking the domain-specific MoA Ontology entities at the top of the hierarchy to appropriate broader entities from Basic Formal Ontology. 

### Step 3 - Refining the MoA Ontology through annotations of MoAs in behaviour change intervention reports

To ensure that the MoA Ontology is clear and aligned with its intended scope, the preliminary ontology was applied to annotate (i.e., code) MoAs reported in published behaviour change intervention evaluations. Two to three researchers independently applied the ontology to annotate 135 intervention evaluation reports on the web-based software, EPPI-Reviewer v4 ( Thomas *et al.*, 2010; Thomas *et al.*, 2020). To help researchers consistently apply the ontology, information on when to annotate MoAs was provided in an ***annotation guidance manual*** ( Wright *et al.*, 2020). The researchers compared their annotations for each paper, and annotation disagreements were discussed to update the ontology’s entity labels, definitions, structure and annotation manual, where relevant.

One hundred and thirty-five reports were annotated, the number informed by two criteria: feasibility to annotate and sufficient scope of papers to refine the ontology ( Wright *et al.*, 2020). Of these reports, 115 were identified by searching key terms (e.g., “behaviour”, “mechanism of action” or “influence” and “theor*”) in two databases: COCHRANE Central and Web of Science. The details of the method to identify, screen and select the 115 reports is presented in https://osf.io/z2cgb. The selected reports were relevant to 30 different target behaviours. The remaining 20 reports were included from a systematic review on effective communication strategies targeting changes in behaviour relevant to infectious diseases (see details on the search strategy in Grimani *et al.* [2021]). Details of the 135 reports can be found in https://osf.io/gufcz.

### Step 4 – Expert stakeholder review of the MoA Ontology

A stakeholder review of the MoA Ontology resulting from Step 3 was conducted to establish that it reflected broader scientific consensus in the behaviour change field and met the needs of potential ontology users ( OBO Foundry, 2019; Wright *et al.*, 2020). Because the MoA Ontology had a large number of entities, and participants were asked to review every entity, it was recognised that the review would be time-consuming for the experts. Therefore, potential participants were offered an honorarium of £650 (£50 per hour x 13 hours). Given the MoA Ontology included entities relevant to a variety of MoAs, we recruited 10 participants with broad theoretical knowledge and expertise in the behaviour change field. The inclusion/exclusion criteria were:

1. Holding a doctoral level degree in a relevant field (psychology, neuroscience, economics, sociology or anthropology)
2. Having at least three relevant and recent (within the last five years prior to recruitment) publications as lead, second or corresponding author on papers about (a) developing, refining or evaluating theories of behaviour or behaviour change, (b) behaviour change interventions applying theories, or (c) reviews of such interventions
3. Not being a close collaborator of the MoA Ontology’s lead developers (AW, JH, PS, RW & SM). Being a close collaborator was defined as: (a) co-authoring a publication three years prior to recruitment (2018 or later) or (b) working for the same institution

***Recruitment***

Three recruitment strategies were used to identify participants. First, an invitation to the review was posted on social media. Secondly, relevant individuals were identified from the authors of book chapters. This strategy involved:

1. Searching for relevant books using broad key terms (“behavio*”, “intervention” and “theory” or “model”) in the search engine GoogleBooks
2. Ordering the identified records according to publication dates, as more recent books were more likely to have authors currently working in behavioural sciences
3. Screening sets of 50 books against exclusion criteria (e.g., textbooks aimed at undergraduate students, no authors specified for chapters) until at least 20 books qualified for further screening
4. Screening book chapter titles and abstracts or introductions for at least one mention of: (1) a human behaviour and (2) a theory or potential MoA with an influence on behaviour
5. Including eligible chapters in a full-text screening to verify at least one mention of: (1) a human behaviour and (2) a potential MoA that influenced behaviour

Thirdly, potential participants were identified from the authors of reviews on MoAs and behavioural theories that were published in two journals: Health Psychology Review and Annual Reviews of Psychology. These journals were selected as they include broad reviews of behavioural theories and interventions. From these journals, participants were identified through the following steps:

1. Searching for key terms (“behavio*”, “intervention” and “theory” or “model”) anywhere in the text of reports that were published in the two journals
2. Randomly selecting 100 reports from the reports identified from each journal
3. Screening report titles and abstracts against inclusion criteria, requiring the report to:
  a. Suggest being a narrative, literature, scoping, conceptual or systematic review, meta-analysis, theory/model building or integration or conceptual critique
  b. Mention an intervention delivered to a human population
  c. Mention a human behaviour
  d. Mention a theory, theoretical framework, model or MoA relevant to behaviour
4. Screening the full text of eligible reports against the same criteria used in the title and abstract screening

The list of authors identified from chapter and review screenings were combined and de-duplicated. In addition, any authors who published papers with the lead ontology developers (since 2018) were removed from the list, leaving 525 potential participants. From this list, 150 participants were selectively approached in order to include participants from diverse countries (see full details of the recruitment strategies reported in https://osf.io/5wq4m).

***Participant screening, procedure and analysis***

Of 15 potential participants expressing interest in the review, 10 met the eligibility criteria and were invited to take part. Before starting the task, they watched short introductory videos, providing an overview of MoAs and the ontology. The expert review was conducted using Qualtrics software (see complete survey here) and included open-ended and closed questions on:

1) Clarity: whether the entity labels and definitions of the ontology can be understood by experts who did not participate in its development
2) Representativeness: whether the ontology comprehensively covers the concepts of interest, i.e., if any entities are missing

To allow participants to refer to the whole ontology at once during the review task, they were also sent a copy of the ontology as a spreadsheet and diagram.

Feedback was extracted from Qualtrics and logged. The ontology development team discussed each issue raised by participants and decided what action to take if necessary. The log was updated with how the ontology was revised to address the feedback or the rationale for not updating the ontology based on that piece of feedback. Where required, the MoA Ontology was revised. For entities reused from the Emotion and Mental Functioning Ontologies ( Hastings *et al.*, 2011; Hastings *et al.*, 2012), changes were negotiated with the HBCP’s ontology expert (JH), who was a developer of the MoA Ontology, as well as the two related ontologies. Based on agreed updates, changes were made to all three ontologies: the MoA, Emotion and Mental Functioning Ontologies.

### Step 5 – Testing the inter-rater reliability of researchers applying the revised version of the MoA Ontology

To ensure that the MoA Ontology’s entity labels and definitions can be reliably applied, we evaluated researchers’ inter-rater reliability in identifying the presence of the ontology’s entities in 100 intervention reports. The method for identifying suitable papers to annotate can be found here, and the full list of reports annotated here. The 100 reports featured interventions targeting 29 different behaviours.

The inter-rater reliability testing was done in two rounds. First, the two researchers leading the ontology’s development applied the ontology to each annotate MoAs in 50 intervention evaluation reports using EPPI-Reviewer software ( Thomas *et al.*, 2010). This number of papers was selected as 50 papers give an accepted 10–15% margin of error around the estimated percentage agreement when calculating inter-rater reliability ( Gwet, 2014). After Round 1, the annotation guidance manual and ontology were updated to tackle any issues that had led to disagreement between coders. In Round 2, inter-rater reliability was assessed for annotations by two researchers unfamiliar with the ontology but with Master’s degrees relating to behaviour change. Inter-rater reliability was assessed using Krippendorff’s alpha ( Hayes & Krippendorff, 2007) calculated using the Automation Inter-Rater Reliability script developed by the HBCP ( Finnerty & Moore, 2020), incorporating the Python script Krippendorff 0.3.2 (April 2019 – January 2021).

Krippendorff’s alpha values above 0.67 are considered to indicate acceptable inter-rater reliability, while values below this threshold can suggest that researchers interpreted the ontology entity labels and definitions differently ( Gwet, 2014; Krippendorff, 2009; Krippendorff, 2011). If the overall Krippendorff’s alpha value was lower than 0.67 for the annotations in a round, the inter-rater reliability of annotations for each entity across the 50 reports was examined. For individual entities with Krippendorff’s alpha values lower than 0.67, all disagreements were reviewed in the relevant intervention reports. The ontology development team discussed and decided upon required changes to the MoA Ontology or annotation manual.

### Step 6 – Specifying the relationships between MoA Ontology entities

The research team discussed and specified the relationships between the MoA Ontology’s entities, some of which had been proposed and refined in Steps 1-5. These included common relationships (e.g., “is_a” and “has_part”) from Basic Formal Ontology and the ***Relation Ontology*** ( Smith *et al.*, 2005). For example, the basic hierarchical relationship “is_a” could be specified between each entity and its parent class. The upper-level entities of the MoA Ontology were also reviewed and a relationship to the broad entity “Behaviour Change Intervention Mechanism of Action” was specified. When necessary, new entities and relationships were developed to structure the ontology through discussions between the research team.

### Step 7 – Reviewing the MoA Ontology’s alignment with other parts of the BCIO and relevant ontologies

The entities and relationships in the final version of the MoA Ontology were reviewed for consistency with other parts of the BCIO and related ontologies that were structured using Basic Formal Ontology: Addiction Ontology ( Hastings *et al.*, 2020), the Emotion Ontology ( Hastings *et al.*, 2011), the Gene Ontology ( Ashburner *et al.*, 2000), Mental Functioning Ontology ( Hastings *et al.*, 2012) and the Ontology of Physical Activity ( Carlier *et al.*, 2022). This review was led by the HBCP’s ontology expert (JH), who flagged inconsistencies between these ontologies. These inconsistencies were discussed with the wider research team and the MoA Ontology or other ontologies were updated as appropriate.

### Step 8 – Reviewing the MoA Ontology’s alignment with the Theories and Techniques Tool

A mapping exercise was conducted to ensure that the MoA Ontology aligned with the 26 MoA groups of the Theories and Techniques (TaT) Tool ( Connell *et al.*, 2019; Johnston *et al.*, 2018; Michie *et al.*, 2018) which included the widely used 14 MoAs of the Theoretical Domains Framework ( Cane *et al.*, 2012; Dyson & Cowdell, 2021; Michie *et al.*, 2005). Two researchers reviewed the entity labels and definitions in the MoA Ontology and recorded which entities were captured by each MoA group in the TaT Tool. Disagreements were discussed and reconciled. The wider research team then reviewed these results and discussed whether additional entities from the ontology or new entities were needed to clearly capture any groups. For new entities, their labels and definitions were drafted and reviewed by the research team.

### Step 9 – Making the MoA Ontology machine-readable and available online

The MoA Ontology was developed as a spreadsheet of entities, with separate rows for each entity with its primary label and definition, and where relevant synonyms, examples and relationships. When the content of the ontology was ready for its initial release, this content was automatically converted into ***Web Ontology Language (OWL)*** ( Antoniou & van Harmelen, 2004) format, enabling it to be viewed and visualised using ontology software such as Protégé ( Musen, 2015) and to be compatible with other ontologies. The conversion to OWL used the ***ROBOT*** ontology toolkit library ( Jackson *et al.*, 2019), which provides a facility to create well-formatted ontologies from spreadsheet-format templates. A ROBOT template is a comma-separated values (CSV) file that can be prepared easily in common spreadsheet software for translation from spreadsheet columns to OWL language and metadata attributes. Within the input template spreadsheet, separate columns represent the entity’s unique alphanumeric identifier (e.g., BCIO:01023), label, definition, relationship with other entities, examples and synonyms. The OWL version of the MoA Ontology was stored on the project’s ***GitHub*** repository, which supports ***versioning*** the ontology (i.e., keeping a record of different versions of the ontology, as updates are made). GitHub also has an ***issue tracker***, which allows feedback to be submitted by the ontology’s users that can be addressed in subsequent releases.

## Results

**Figure 4.**
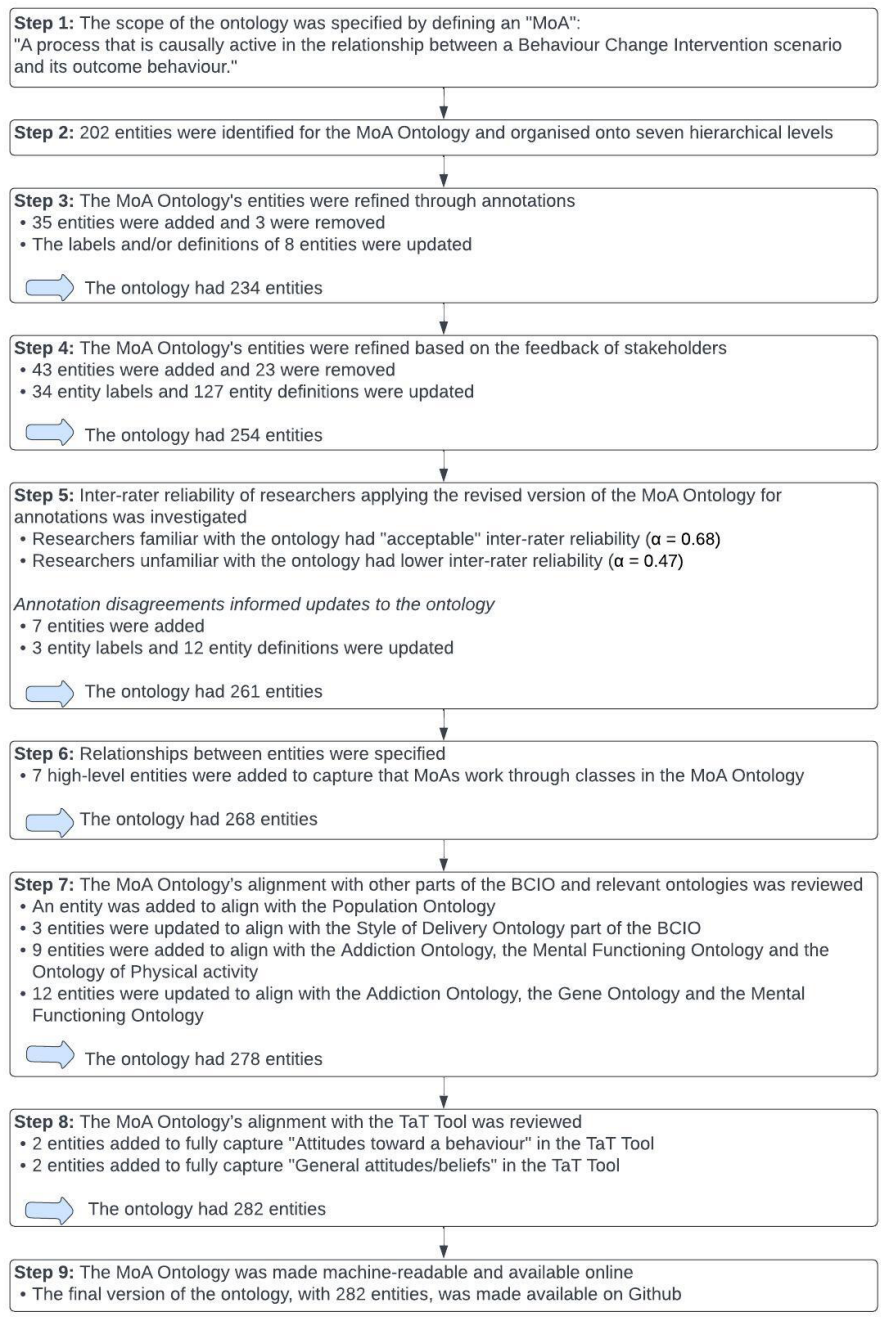
Summary of results from each step to develop the MoA Ontol.

The results from each step to develop the MoA Ontology are summarised in Figure 4.

### Step 1 – Defining the scope of the MoA Ontology

A “Behaviour Change Intervention MoA” was defined as “A process that is causally active in the relationship between a Behaviour Change Intervention scenario and its outcome behaviour.” Every entity in the MoA Ontology can be thought of as an entity (a thing, tangible or intangible, object or process) that an intervention’s MoA works through. For instance, the ontology includes an entity labelled as “belief”. An intervention could work by changing someone’s belief (MoA) or heightening the salience of an existing belief and thereby changing their behaviour. In formal terms, we would describe this intervention’s MoA as: “MoA through belief”.

### Step 2 – Identifying, labelling and defining entities for the MoA Ontology

Of the 1733 theoretical constructs, 1062 were judged to qualify as potential MoAs. Of these MoAs, 146 were judged to be compound MoAs and so excluded from the grouping task (see list of constructs here: https://osf.io/ze6g4). Altogether, 763 MoAs were organised into 104 MoA groups and 153 MoAs remained ungrouped (see https://osf.io/ze6g4). Examples of MoA groups included “Self-efficacy” (N= 49 MoAs), “Intention” (N= 24), “Knowledge” (N= 23) and “Social influence” (N= 12).

Drawing on the 104 groups and the 153 ungrouped MoAs and reusing entities from other ontologies where appropriate, 202 entities were identified and organised on seven hierarchical levels (see https://osf.io/tgkme) An example of the hierarchically organised entities is shown in Table 3.

**Table 3.** Example of hierarchically organised ontology entities created in Step 2.

Level 1	Level 2	Level 3	Definition
belief			A disposition to mental processes that represent some proposition X to be true.
	belief about barriers		A belief about the extent to which factors exist that could restrict or impede the person from engaging in a behaviour.
	belief about conformity to behavioural norms		A belief about the extent to which one's own behaviour is similar to that of referent others.
	belief about consequences of behaviour		A belief about the outcomes resulting from a behaviour.
		belief about social consequences of behaviour	A belief about the outcomes of a behaviour in terms of social processes or attributes.

### Step 3 - Refining the MoA Ontology through annotations of MoAs in behaviour change intervention reports

Based on the annotations with the MoA Ontology resulting from Step 2, 184 issues were recorded and responded to (see https://osf.io/n2qvh). In response to these issues, 35 entities were added to the MoA Ontology and the labels and/or definitions of eight entities were updated. For instance, in an intervention report, an MoA was labelled as “guilt” ( Rupp *et al.*, 2019), and the researchers found the entity “emotion process” too broad for this MoA. Therefore, to capture this MoA more clearly, the entity “guilt” was added to the ontology. Eight ***domain-neutral entities***, i.e., not specific to a scientific domain (e.g., “disposition”), were also no longer shown as part of the ontology, as they were considered too broad to capture MoAs in intervention reports. At the end of Step 3, the ontology had 229 entities.

### Step 4 – Expert stakeholder review of the MoA Ontology

Of the 10 participants in the stakeholder review, nine completed the review. These nine participants worked in institutions based in the following countries: Australia (n = 1), Canada (n = 1), France (n = 1), Ireland (n = 1), United Kingdom (n = 3) and United States (n = 2).

Participants suggested that 61 entity labels and 195 entity definitions needed changing (see https://osf.io/9fmyu). Participants made 606 comments outlining issues with a specific entity or with the ontology more generally. Each comment was responded to by the research team to explain steps to address the issue or the rationale for not revising the ontology in response to that comment (see https://osf.io/82g9c). Based on the expert stakeholders’ comments, the research team updated 34 entity labels, 127 entity definitions and the parent classes of 25 entities. For example, one participant indicated that the “mental process” definition (“A bodily process that is of a type such that it can of itself be conscious”) was underspecified, while another participant pointed out that mental processes do not always involve consciousness. To better specify this entity, the definition was updated to “A bodily process that occurs in the brain, and that can of itself be conscious, or can give rise to a process that can of itself be conscious or can give rise to behaviour.” Eighteen entities were considered captured by other entities or too granular and so were removed from the ontology. Moreover, 43 entities were added to the ontology, which then had 254 entities. To support a better understanding of some entities, the research team also added comments to 59 entities, synonyms to 11 entities and examples to three entities.

### Step 5 - Testing the inter-rater reliability of researchers applying the revised version of the MoA Ontology

The two researchers familiar with the MoA Ontology had an “acceptable” inter-rater reliability (α = 0.68) for annotations using the ontology. The inter-rater reliability of annotations by researchers unfamiliar with the ontology was lower (α = 0.47). The inter-rater reliability for each annotated entity across the 50 reports for each set of researchers is shown in: https://osf.io/tgxey (Round 1) and https://osf.io/hjmxb (Round 2). 

As the Krippendorff’s alpha was above 0.67 for Round 1 annotations, the disagreements were not systematically analysed. However, minor changes were made to the ontology and guidance based on issues noted by the annotators (see https://osf.io/drtgm). For instance, four entities (e.g., “feeling at ease”) were added to the ontology to capture specific MoAs in intervention reports.

Given that the overall Krippendorff’s alpha was below 0.67 for Round 2, alpha values were examined at the level of individual entities. For each entity where Krippendorff’s alpha was below 0.67, all annotation disagreements were reviewed. The ontology developers either took steps to address these disagreements by revising the ontology and its associated annotation guidance or recorded the rationale for not revising the ontology. For example, a disagreement might not lead to a change in the ontology if the disagreement was judged to be due to insufficient detail about the MoA in the original paper. A log was kept of all decisions (see https://osf.io/79gav).

After examining researchers’ annotation disagreements in Round 2, three entities were added, and three entity labels, 12 entity definitions and informal definitions of two entities were updated. Comments were added to seven entities and an example was added to one entity. This process resulted in an ontology with 261 entities. There were seven upper-level entities in the ontology: “material entity”, “environmental disposition”, “location”, “bodily disposition”, “cognitive representation”, “personal role” and “bodily process”.

### Step 6 – Specifying the relationships between MoA Ontology entities

For entities in the MoA Ontology, relationships from the Relation Ontology ( Smith *et al.*, 2005) were used to link entities together. Each entity was linked to a parent class using the hierarchical relationship “is_a”. For instance, the entity “belief about one’s environment” was linked to its parent class “belief”. In the ontology, this relationship was specified as: “belief about one’s environment” is_a “belief”. Some entities were specified as being “part_of” another entity, e.g., “self-efficacy belief for a behaviour” is part of “self-efficacy belief for a behaviour and its associated outcomes”.

The structure of the MoA Ontology also needed to capture that MoAs work through entities in this ontology. Entities in the MoA Ontology (e.g., “happiness”) are not MoAs by themselves, but are entities (e.g., processes) through which intervention MoAs can work. For instance, a person can experience happiness in the absence of any intervention, e.g., when walking by the seaside. The entity “happiness” would only be part of an MoA if an intervention works through changing, or changing the salience of, “happiness” to bring about behaviour change.

**Figure 5.**
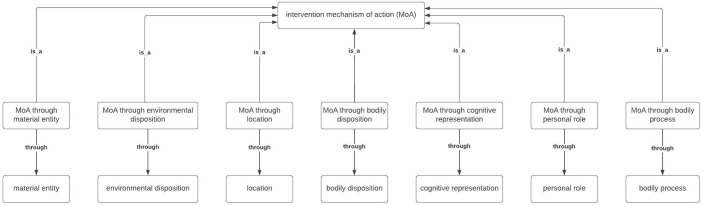
The 7 upper-level entities in the MoA Ontology.

To capture that MoAs work through “entity x”, the seven upper-level entities in the MoA Ontology (e.g., “bodily disposition”) needed to be formally linked within the ontology to new entities specified as “MoA through entity x”. Therefore, seven entities, one corresponding to each of the upper-level entities, were added to the MoA Ontology (e.g., “MoA through bodily disposition”). The “through” relationship between “MoA through entity x” and the relevant “entity x” was specified (see Figure 5). Each lower-level entity was assumed to adopt this formulation (“MoA through entity x”) through their hierarchical relationships to the seven upper-level entities. For instance, the lower-level entity “belief” in the MoA Ontology should be taken to imply an MoA of the form “MoA through belief”, which captures that a belief is an intervention’s MoA when it is targeted by an intervention to bring about the intervention’s influence on behaviour.

As the “through” relationship was specific to MoAs in behaviour change interventions, there was no relevant relationship that could be reused from the Relation Ontology ( Smith *et al.*, 2005). Instead, the research team defined this relationship as “A relationship that holds between an intervention’s MoA and an entity x, in which the entity x (a) participates in or is part of the MoA process and (b) is influenced by a behaviour change intervention or its context such that there is some change in entity x.” In the definition, “change” refers to change from what would have been the case rather than change from an existing state of affairs. This is to capture the fact that MoAs can act to sustain a current state of affairs, for example maintaining motivation not to smoke. In the definition, “some change” captures changes in salience, change in valence, or being added, increased, decreased, manifested/realised, created, started, stopped or altered in rate. Comments were added to the seven upper-level entities (e.g., “MoA through bodily disposition”) noting the nature of this relationship.

### Step 7 – Reviewing the MoA Ontology’s alignment with other parts of the BCIO and relevant ontologies

In the review of the MoA Ontology’s alignment with other parts of the BCIO, one entity (“belief about quality of life”) was added and three entities were identified as requiring updates. For instance, the label of “communication behaviour” was updated to be more clearly about human communication (“human communication behaviour”), thereby better aligning with the Style of Delivery Ontology (under development at time of writing).

Following the review of the alignment between the MoA Ontology and five relevant external ontologies (Addiction Ontology, Emotion Ontology, Gene Ontology, Mental Functioning Ontology, the Ontology of Physical Activity), 12 entities were updated, and nine were added to the MoA Ontology. For instance, the “identity” and “group identity” entities were added to better align with the Addiction Ontology, and three entities relating to goal interaction (e.g., “goal conflict”) were added to link better to the Ontology of Physical Activity. The resulting MoA Ontology had 278 entities. Other discrepancies were resolved by updating entities in the Addiction, Emotion and Mental Functioning Ontologies.

### Step 8 – Reviewing the MoA Ontology’s alignment with the Theories and Techniques Tool

Two researchers’ initial mapping of the MoA Ontology entities onto the Theory and Techniques Project’s 26 MoA groups can be found in the following link: https://osf.io/ycdzv. After discussions with the wider ontology team, two new entities (e.g., “affective attitude”) were added to fully capture the MoA group “Attitude towards a behaviour” and two were added to better capture “General Beliefs/Attitude”, resulting in 282 entities in the ontology. In total, 46 entities (not counting their subclasses) were mapped onto the 26 MoAs (1-4 entities per MoA group). The final mapping is at: https://osf.io/zmub5.

### Step 9 - Making the MoA Ontology machine-readable and available online

The final version of the MoA Ontology has 282 entities and is downloadable from GitHub. The ontology’s hierarchical structure, alphanumeric identifiers, labels, definitions, synonyms, comments and examples of all entities are at: https://osf.io/pkq4e. The ontology is accompanied by an annotation guidance manual, revised based on the results of Step 5, that provides guidance on how to annotate these entities in behaviour change intervention reports (available at https://osf.io/um7w6).

## Discussion

An MoA Ontology has been developed within the Behaviour Change Intervention Ontology consisting of 282 entities, organised in seven hierarchical levels. The upper-level entities are: “MoA through material entity”, “MoA through environmental disposition”, “MoA through location”, “MoA through bodily disposition”, “MoA through cognitive representation”, “MoA through personal role” and “MoA through bodily process”. This method of specifying MoAs in terms of entities, labels and definitions provides a shared vocabulary for reporting theoretical processes of change in interventions and their evaluations. This increases clarity, reduces ambiguity and enables communication across disciplines and theoretical orientations about MoAs.

The MoA Ontology can be used to support evidence syntheses about MoAs and integration of MoAs themselves, by specifying and categorising them precisely. This can inform the selection of MoAs to target for changing particular behaviours, thereby enabling the development of more effective interventions, and inform the development and refinement of behavioural theories. For instance, by drawing on evidence that certain MoAs are consistently not associated with behaviour change, theory authors could remove relevant constructs from their theories. The ontology can also be used to map evidence about MoAs onto its entities, as part of creating an “evidence-gap map” ( Britton *et al.*, 2021). Such maps can help researchers identify MoAs that require more study and avoid repeatedly investigating the same MoAs.

Inter-rater reliability for annotating research reports using the ontology was “acceptable” (α = 0.68) for researchers familiar with the MoA Ontology, but lower for those unfamiliar with the ontology (α = 0.47). This suggests that more guidance and training for using the MoA Ontology will be needed for those unfamiliar with intervention annotation or with ontologies; the development of training is in progress and can be found on https://www.bciontology.org/.

The MoA Ontology is connected to other ontologies that form part of the BCIO ( Michie *et al.*, 2021), such as the Behaviour Change Techniques, Intervention Source, Mode of Delivery and Setting Ontologies ( Marques *et al.*, 2021; Marques *et al.*, 2023; Norris *et al.*, 2020; Norris *et al.*, 2021b). These ontologies can be used together to synthesise detailed evidence about various aspects of behaviour change interventions ( Michie *et al.*, 2017), to design interventions and to plan their evaluation. For example, the Behaviour Change Techniques Ontology ( Marques *et al.*, 2023) and the MoA Ontology can be used together to capture interventions’ content and their mechanisms of action. The entities in the MoA Ontology can also be linked and reused by ontologies beyond the BCIO, such as the Emotion Ontology ( Hastings *et al.*, 2011), Mental Functioning Ontology ( Hastings *et al.*, 2012), Gene Ontology ( Ashburner *et al.*, 2000), the Ontology of Medically related Social Entities ( Hicks *et al.*, 2016) and the Addiction Ontology ( Hastings *et al.*, 2020).

In parallel to the work developing the MoA Ontology, an Ontology-based Modelling System (OBMS) was developed and applied to precisely represent 76 behavioural theories by labelling constructs (“entities”) and specifying their relationships ( Hale *et al.*, 2020b; West *et al.*, 2019). By mapping the constructs that influence behaviour and through which pathways (directly or through other constructs), these formal representations help select potential MoAs for behaviour change interventions. By virtue of the theory representations being computer readable, a study is currently investigating the integration of these representations into one or more “canonical” theories. While this Ontology-based Modelling System outlines explicit relationships between constructs for each theory reviewed ( Hale *et al.*, 2020b), the MoA Ontology provides a formal language for MoAs not restricted to particular theories. Both reduce ambiguity about constructs and relationships that can arise from using very varied “natural language” in theory descriptions.

### Strengths and limitations

A key strength of this study was the systematic approach to ontology development, including the formal step of integrating feedback from different domain experts to provide a range of perspectives to test the ontology’s accuracy and relevance to its users ( Amith *et al.*, 2018). Many ontologies are developed without such an explicit, formal stakeholder review ( Norris *et al.*, 2019; Norris *et al.*, 2021a). Another strength of the MoA Ontology’s development was structuring the ontology using Basic Formal Ontology (BFO), which enabled close collaboration with developers of related ontologies that also drew on BFO: Addiction, Emotion and Mental Functioning Ontologies ( Hastings *et al.*, 2011; Hastings *et al.*, 2012; Hastings *et al.*, 2020). Entities in these ontologies were reused where possible, making the MoA Ontology interoperable with these ontologies, and reducing the duplication. Moreover, refining the MoA Ontology helped improve relevant entities in these related ontologies. For instance, based on the stakeholder review of the MoA Ontology, the definitions of various entities reused from the Emotion and Mental Functioning Ontologies ( Hastings *et al.*, 2011; Hastings *et al.*, 2012) were refined to be clearer in these ontologies.

By identifying entities from behavioural theories, intervention reports and stakeholder feedback, this study drew on diverse sources to include a wide range of MoAs to get wide coverage of the domain of interest. In parallel, efforts were made (Step 3 and 4) to remove entities that added detail to the ontology without capturing key distinctions between MoAs. Applying the MoA Ontology to annotate MoAs in intervention reports, and accordingly refining its entity labels and definitions, improved the ontology’s clarity ( Amith *et al.*, 2018; Wright *et al.*, 2020). With its 282 entities, the MoA Ontology is one of the larger and more complex ontologies that forms part of the BCIO (for other parts see e.g., Marques *et al.*, 2021; Norris *et al.*, 2020; Norris *et al.*, 2021b).

This study found that new users of the MoA Ontology had challenges in reliably applying the ontology. While one reason for this finding may be the complexity of the MoA Ontology, lack of specificity in the reporting of MoAs in papers also reduced inter-rater agreement. For instance, many intervention reports do not (1) clearly specify the intervention’s intended MoAs (e.g., the relationship between constructs and behaviour), (2) provide definitions for MoAs or (3) in the absence of MoA definitions, provide detail of measurement items for each MoA. By improving the reporting of MoAs in intervention reports and applying the MoA Ontology to help synthesise evidence about MoAs, the evidence base regarding MoAs can become more reliable. In addition, the annotation manual can support ontology users when applying this ontology to code MoAs in intervention reports. We are developing training resources and visualisation tools to support users applying the ontology to their projects. In line with good practice in ontology development, we expect that the MoA Ontology will be updated and revised based on the feedback from a wide range of users, improving the ontology’s applicability ( Arp *et al.*, 2015; He *et al.*, 2018). Potential users can access the ontology through GitHub and provide feedback on the ontology by creating an “Issue” on this portal. For instance, if more detailed entities are needed in the ontology, users can suggest these as new entities on GitHub and these can be added to the ontology by the developers. Guidance on how to do this can be found on BCIO. In addition to GitHub, the up-to-date version of the MoA Ontology will be available on via BCIOSearch, Ontology Lookup Service (OLS) tools and the Behavioural and Social Sciences Ontology Foundry repository.

## Conclusion

The Behaviour Change Intervention MoA Ontology provides a detailed classification system that labels and defines entities to describe MoAs of behaviour change interventions. This ontology can support more accurate and consistent reporting and efficient evidence synthesis about MoAs across different interventions (e.g., in systematic reviews). It can also link bodies of knowledge across theories, topic domains, academic disciplines and types of knowledge. Further, by being a computer-readable classification system, this ontology can be used to build computational tools to automatically extract information about MoAs (e.g., from intervention reports) and use knowledge from intervention evaluations to make predictions about MoAs ( Michie *et al.*, 2017). The ontology can be further extended and refined through users’ feedback, and thereby become an increasingly useful resource for improving understanding about “why” behaviour change interventions work or do not.

## Ethics

Ethical approval was granted by University College London’s ethics committee (CEHP/2020/579). Participant consent was gained in the stakeholder review on a page of the online Qualtrics survey.

## Data Availability

Open Science Framework: Human Behaviour-Change Project, https://doi.org/10.17605/OSF.IO/QRGC4 ( West *et al.*, 2020). This project contains the following underlying data: Expert feedback on Mechanism of Action Ontology; Raw feedback received from behavioural science experts.pdf, https://osf.io/82g9c Open Science Framework: Human Behaviour-Change Project, https://doi.org/10.17605/OSF.IO/QRGC4 ( West *et al.*, 2020). This project contains the following extended data: The detailed guidelines for identifying mechanism of action from the constructs of behavioural theories; https://osf.io/9j2be The theoretical constructs judged to be compound mechanisms of action and not included as entities in the Mechanism of Action Ontology; https://osf.io/ze6g4 The theoretical constructs identified as mechanisms of action and, where relevant, their initial groupings; https://osf.io/ze6g4 The entities hierarchically organised in the initial version of the Mechanism of Action Ontology; https://osf.io/tgkme The details of the method to identify papers to annotate mechanisms of action with the Mechanism of Action Ontology; https://osf.io/z2cgb Papers used in development of the Mechanism of Ontology in Step 3 to refine the ontology; https://osf.io/gufcz The issues recorded when applying the Mechanism of Action Ontology to annotate mechanisms of action in interventions reports in Step 3 and responses to these issues; https://osf.io/n2qvh The details of the method to recruit participants for the stakeholder review of the Mechanism of Action Ontology; https://osf.io/5wq4m Expert feedback survey; Full survey provided to behaviour science experts in the review of the Mechanism of Action Ontology; https://osf.io/ycd73 Mechanism of Action Ontology entity labels and definitions identified as requiring changing in the stakeholder review of the ontology; https://osf.io/9fmyu Papers used in development of the Mechanism of Ontology in Step 5 to test inter-rater reliability using the ontology; https://osf.io/sjd2b Inter-rater reliability testing for annotations by researchers familiar with the Mechanism of Action Ontology; https://osf.io/tgxey The issues recorded by researchers familiar with the Mechanism of Action Ontology when applying it to annotate mechanisms of action in interventions reports in Step 5 and responses to these issues; https://osf.io/drtgm Inter-rater reliability testing for annotations by researchers unfamiliar with the Mechanism of Action Ontology; https://osf.io/hjmxb Annotation disagreements between researchers unfamiliar with the Mechanism of Action Ontology and log of decisions to address these disagreements: https://osf.io/79gav Coding guidelines; Manual for coding using the Mechanism of Action Ontology; https://osf.io/um7w6 The initial mapping of the MoA Ontology entities onto the TaT Project’s MoA groups; https://osf.io/ycdzv The final mapping of the MoA Ontology entities onto the TaT Project’s MoA groups; https://osf.io/zmub5 The first complete version of the MoA Ontology; https://osf.io/pkq4e OSF page for the Human Behaviour-Change Project; Homepage for all outputs across the project; https://osf.io/h4sdy/ Zenodo: HumanBehaviourChangeProject/ontologies: HumanBehaviourChangeProject/ontologies: Upper-Level, Setting, Mode of Delivery & Source ontologies. https://zenodo.org/record/4476603#.YBLtcOj7SUk ( Hastings *et al.*, 2021) Data are available under the terms of the Creative Commons Attribution 4.0 International license (CC-BY 4.0).

## Software availability

Source code used to calculate alpha for IRR available from: https://github.com/HumanBehaviourChangeProject/Automation-InterRater-Reliability.

Archived code at time of publication: https://doi.org/10.5281/zenodo.3833816 ( Finnerty & Moore, 2020)

License: GNU General Public License v3.0 only
