# Supplementary material for: An ontology of mechanisms of action in behaviour change interventions
Source: Wellcome Open Res. 2024 May 30;8:337. Originally published 2023 Aug 11. [Version 2] doi: 10.12688/wellcomeopenres.19489.2 (PMC10933577; doi:10.12688/wellcomeopenres.19489.2)
Supplement: Peer review history

# Reviewer report: Reviewer response for version 2

**DOI:** 10.21956/wellcomeopenres.24763.r103915

**Published:** 2024-11-01

**Authors:** Helen Stringer

**Affiliations:** 1 Newcastle University, Newcastle upon Tyne, England, UK

I welcome this paper and the detail of Mechanisms of Action (MoA) in behaviour change interventions. MoA can be a concept difficult to explain, especially when desired behaviours are actively maintained rather than overtly changed. This paper makes MoA very clear throughout, with helpful examples. Training to improve inter-rater reliability is an ongoing issue, addressed here by signposting existing training and future training plans outlined. I expect this to be of a high standard, in line with existing online training for users of the BCIO. The use of existing ontologies in different fields to avoid duplication and the rigorous aligning with other sections of the BCIO and the theory neutral nature of the MoA Ontology is a testament to the authors’ (and wider BCIO team’s) commitment to making the BCIO widely accessible.

 I have read the paper, taking into consideration the previous reviews and consider it to be robust, clear and informative. It is a very useful addition to the BCIO.

Is the work clearly and accurately presented and does it cite the current literature?

Yes

If applicable, is the statistical analysis and its interpretation appropriate?

Yes

Are all the source data underlying the results available to ensure full reproducibility?

Yes

Is the study design appropriate and is the work technically sound?

Yes

Are the conclusions drawn adequately supported by the results?

Yes

Are sufficient details of methods and analysis provided to allow replication by others?

Yes

Reviewer Expertise:

As an academic speech and language therapist, most of my research and teaching focuses on behaviour change mechanisms and techniques. I used the BCTTv1 extensively and am familiar with the BCIO.

I confirm that I have read this submission and believe that I have an appropriate level of expertise to confirm that it is of an acceptable scientific standard.

# Reviewer report: Reviewer response for version 1

**DOI:** 10.21956/wellcomeopenres.21587.r73435

**Published:** 2024-03-12

**Authors:** Paulius Yamin

**Affiliations:** 1 The London School of Economics and Political Science (Ringgold ID: 4905), London, England, UK; 2 Paris Institute for Advanced Study, Paris, France

This paper presents the process and results of an endeavour that is sorely needed in the behavioural intervention / behavioural science fields: a solid classification system for the mechanisms that link behavioural change interventions and their outcome behaviours. The authors present a solid and rigorous process for producing the first encompassing ontology of mechanisms of action in behavioural interventions and describe and discuss the (necessarily) complex results.

 Given (as the authors report) the inconsistency and ambiguity in how these mechanisms are reported in behavioural interventions, I expect this work to have considerable impact in the way we design, report, evaluate and understand behavioural interventions in both academic and applied settings – depending of course on the adoption from both researchers and practitioners of the ontology. If successful, the ontology will (at last) allow us to precisely describe and understand the effects of different interventions across domains, populations, and target behaviours (in which at present, it is often difficult or even impossible to tease out what mechanisms are leveraged exactly, and how they compare to the ones leveraged by other interventions).

 There are two specific elements I'd like to raise because I believe them to be important for the soundness and impact of the proposed framework. These might be better explored in subsequent publications of the project, as I consider them to be relatively complex and they don't impair the overall quality of this paper"

  but that I mention because I believe them to be essential to the soundness and impact of the proposed framework:

- The training / dissemination side of the framework proposed seems to be essential for its impact, not only for its overall adoption but also for the accuracy of its application beyond the researchers that are already familiar with it (addressing the considerably lower inter-reliability scores for intervention annotation from researchers that were not familiar with the ontology). In my opinion, it will be essential to thoroughly test and adjust both the ontology and the dissemination and training materials associated with it to ensure that it is reasonably clear and easy to apply by researchers and practitioners seeking to design and report behavioural interventions.
- I’d be curious to know as well how you deal with the combination of different MoAs in a single intervention, both in the identification / annotation stage, and also in the potential evaluation / prediction work that could be conducted using them. Complex interventions (especially in non-academic settings) will often leverage several MoAs in an attempt to impact behaviour, and there’s an argument to be made that specific combinations can have an exponential effect. Was any information on that collected, or do you plan to collect it in future analyses?

Finally, I also wonder about the large proportion of health-oriented interventions (in a broad sense) that were included in the annotated reports ion step 3. While I believe this is legitimate at this stage, I wonder if you plan to test the ontology in behavioural interventions that are oriented towards a more diverse range of domains that are popular in the literature (e.g. environmental issues, participation, etc).

 Overall, a very strong paper with a framework that I expect (and hope) will have a considerable impact in behavioural interventions across domains.

Is the work clearly and accurately presented and does it cite the current literature?

Yes

If applicable, is the statistical analysis and its interpretation appropriate?

Yes

Are all the source data underlying the results available to ensure full reproducibility?

Yes

Is the study design appropriate and is the work technically sound?

Yes

Are the conclusions drawn adequately supported by the results?

Yes

Are sufficient details of methods and analysis provided to allow replication by others?

Yes

Reviewer Expertise:

Behavioral science, behavioral intervention, social norms, participation,  civic culture, sustainability, collective action

I confirm that I have read this submission and believe that I have an appropriate level of expertise to confirm that it is of an acceptable scientific standard.

# Reviewer report: Reviewer response for version 1

**DOI:** 10.21956/wellcomeopenres.21587.r65258

**Published:** 2023-10-12

**Authors:** Richard Cooke

**Affiliations:** 1 School of Health, Wellbeing and Science, Staffordshire University, Stoke-on-Trent, England, UK

The authors report results of a series of studies conducted to develop the Mechanisms of Action Ontology. The paper is well-written and of interest to researchers working to better understand why interventions work. The two questions I had after reading the paper, both related to Figure 5.

 First, I may have missed this, but I was unsure where the seven upper-level entitites came from; the top of page 14 states there were seven upper-level entities but does not specify how these entities emerged from the process of ontology development. How did the researchers settle on these entities?

 Second, I struggled to see the purpose of Figure 5, which reminds me of a logic diagram used in computer science, where you need to tell a computer something that is immediately obvious to humans. I'm guessing there is something important here but struggle to see what it is.

 Overall, this paper makes a valuable contribution to the literature.

Is the work clearly and accurately presented and does it cite the current literature?

Yes

If applicable, is the statistical analysis and its interpretation appropriate?

Yes

Are all the source data underlying the results available to ensure full reproducibility?

Yes

Is the study design appropriate and is the work technically sound?

Yes

Are the conclusions drawn adequately supported by the results?

Yes

Are sufficient details of methods and analysis provided to allow replication by others?

Yes

Reviewer Expertise:

Health psychology

I confirm that I have read this submission and believe that I have an appropriate level of expertise to confirm that it is of an acceptable scientific standard.
